# Genetic modules for α‐factor pheromone controlled growth regulation of *Saccharomyces cerevisiae*

**DOI:** 10.1002/elsc.202300235

**Published:** 2024-05-22

**Authors:** Uta Gutbier, Juliane Korp, Lennart Scheufler, Kai Ostermann

**Affiliations:** ^1^ Faculty of Biology Research Group Biological Sensor‐Actuator‐Systems TUD Dresden University of Technology Dresden Germany; ^2^ Else Kröner Fresenius Center for Digital Health Faculty of Medicine Carl Gustav Carus TUD Dresden University of Technology Dresden Germany

**Keywords:** growth regulation, pheromone response, *Saccharomyces cerevisiae*, synthetic biology, α‐factor

## Abstract

*Saccharomyces cerevisiae* is a commonly used microorganism in the biotechnological industry. For the industrial heterologous production of compounds, it is of great advantage to work with growth‐controllable yeast strains. In our work, we utilized the natural pheromone system of *S. cerevisiae* and generated a set of different strains possessing an α‐pheromone controllable growth behavior. Naturally, the α‐factor pheromone is involved in communication between haploid *S. cerevisiae* cells. Perception of the pheromone initiates several cellular changes, enabling the cells to prepare for an upcoming mating event. We exploited this natural pheromone response system and developed two different plasmid‐based modules, in which the target genes, *MET15* and *FAR1*, are under control of the α‐factor sensitive *FIG1* promoter for a controlled expression in *S. cerevisiae*. Whereas expression of *MET15* led to a growth induction, *FAR1* expression inhibited growth. The utilization of low copy number or high copy number plasmids for target gene expression and different concentrations of α‐factor allow a finely adjustable control of yeast growth rate.

**Abbreviations**
GPCRG: protein‐coupled receptor
HA_3_
: three copies of the viral hemagglutinin protein tag
Mw calc.: calculated molecular weight
RNU: relative nephelometric units
*S. cerevisiae*
: *Saccharomyces cerevisiae*

## 1 INTRODUCTION

Due to the easy genetic tractability and the extensive knowledge regarding its metabolism, genetics and lifestyle, the yeast *Saccharomyces cerevisiae* is a commonly applied microorganism in the biotechnological industry, for example, for beverage, food or biofuel production [1, 2, 3]. For maintaining stability in biotechnological production cultures or co‐cultures, one critical point is the controllable growth behavior of the microorganisms. In general, yeast growth can be controlled by the availability of nutrients such as sugars, amino acids, or nitrogen compounds, as these determine development programs and growth rates [4]. Different compounds such as farnesol [5], proteinogenic amino acids as well as the nonproteinogenic amino acids citrulline and ornithine [6], or weak acids like acetic acid or formic acid [7] possess growth‐inhibiting effects at high levels. For some applications, the use of specific mutations may also be an alternative constitutive method for growth control, for example, using auxotrophic or temperature‐sensitive strains [8, 9]. However, it is not always possible to change medium composition or use specific *S. cerevisiae* strains to influence the growth rate, especially with regard to co‐cultures or biotechnological production systems. The growth control system we generated differs, since the direct inducibility by the α‐factor creates yeast specificity as well as independency on nutrient availability.

Naturally, cells of *S. cerevisiae* occur in three forms, namely haploid **a**‐cells, possessing the MAT**a** mating type, haploid α‐cells with MATα mating type, and diploid **a**/α‐cells, which emerge from the mating of two compatible haploid cells [10, 11, 12, 13]. The mating process commences with the secretion and perception of mating type specific diffusible pheromones. *S. cerevisiae* **a**‐cells produce the **a‐**factor pheromone [14, 15, 16, 17], which is recognized specifically by the G‐protein‐coupled receptor (GPCR) Ste3p located at the surface of α‐cells [18]. In contrast, α‐cells secrete an unmodified peptide consisting of 13 amino acids termed α‐factor [19, 20, 21]. Perception of α‐factor by **a**‐cells is realized by the Ste2p GPCR [22]. Binding of the pheromones to their specific cell surface receptors initiates the mitogen‐activated protein kinase (MAPK)‐based signal‐response pathway [23, 24, 25, 26]. As a consequence, several cellular changes are induced, including an arrest of the yeast cell cycle in G1, the induction of mating specific genes and morphological changes like the polarized cell growth (Shmoo tip formation) [12, 27, 28].

**FIGURE 1:**
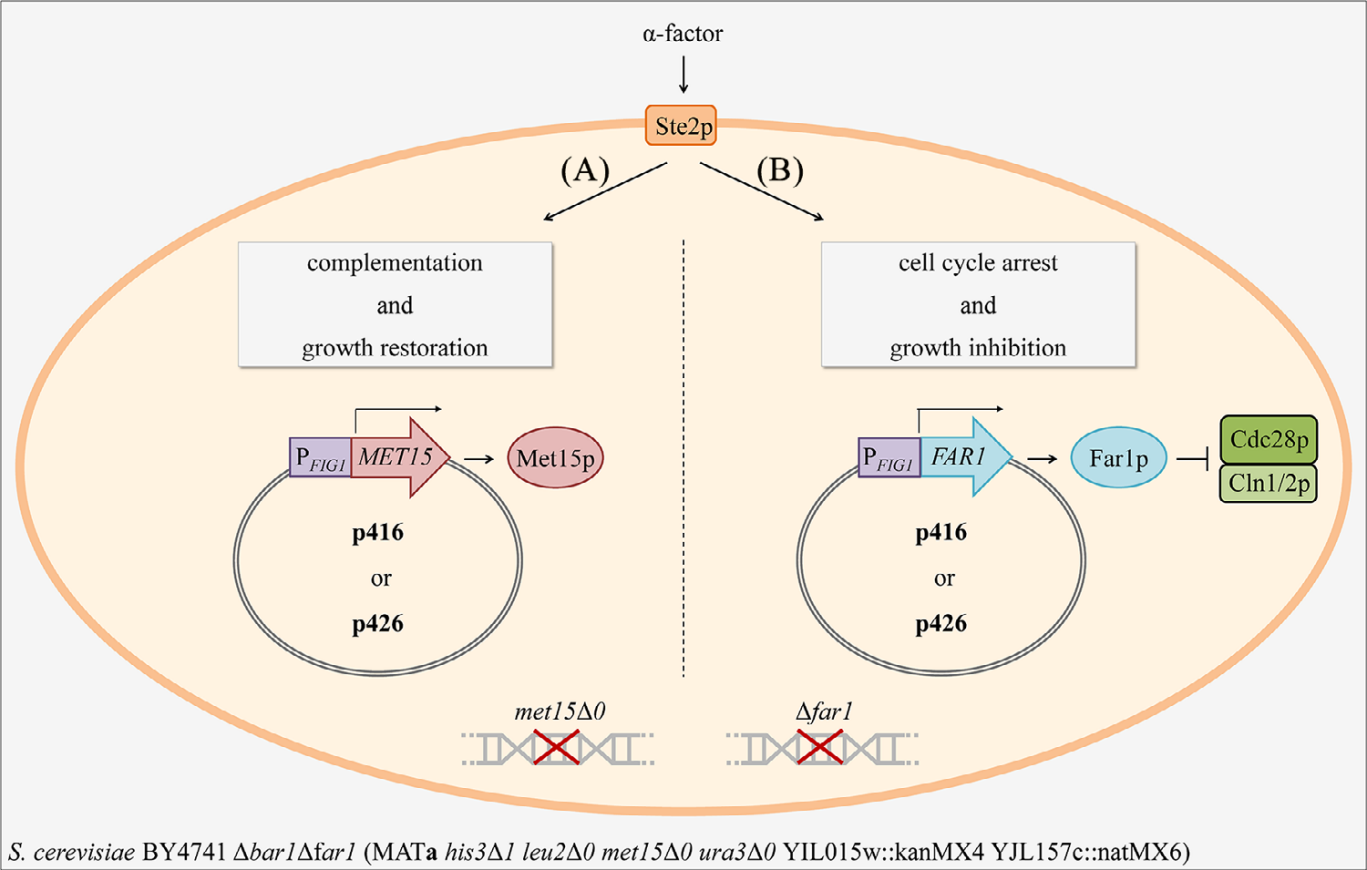
Approach for pheromone induced growth restoration (A) or growth inhibition (B) of *S. cerevisiae* BY4741 Δ*bar1*Δ*far1*. Expression of the target genes *MET15* or *FAR1* was under control of the α‐factor sensitive *FIG1* promoter. Met15p complements the methionine auxotrophy in the expression strain (A), whereas Far1p induces cell cycle arrest by inhibiting the Cdc28p/Cln1/2p complex (B). The p416 low copy number and the p426 high copy number plasmids, which differ in their replication origins (p416: CEN/ARS; p426: 2μ origin), served as vectors [33].

One early target of the pheromone response in *S. cerevisiae* is the *FIG1* gene, whose expression is fast and highly upregulated after pheromone binding [29, 30]. The protein Fig1p functions in cell polarization and the development of the mating projection shape during the mating process [30]. The pheromone‐dependent promoter of *FIG1* has been exploited in previous expression studies, showing its great potential to control gene expression by mating pheromones [31, 32].

**Practical Application**

For biotechnological production systems it is important to keep control over the host organisms in culture. One commonly used microorganism in biotechnology is *Saccharomyces cerevisiae* due to its robustness and its wide application field. In our study, we developed plasmid based genetic modules, which can be activated by the addition of the α‐factor pheromone. Exploiting the natural pheromone signaling cascade of *S. cerevisiae*, we created different strains whose growth is controllable by the addition of α‐factor. This system allows a precise and dynamic, α‐factor‐dependent increase or decrease of the growth rate of the respective strain. Another conceivable use of the yeast strains generated in this study is the co‐cultivation with other microorganisms, resulting in a biosynthetic division of labor and an increased metabolic efficiency. The growth rates of our yeast strains can be modulated, for example, to adjust growth or to prevent overgrowth of an organism in a co‐culture.

*S. cerevisiae* **a**‐cells are known to secrete the protein Bar1p (“barrier” activity), which acts as antagonist of the α‐factor pheromone [33, 34]. In turn, a deletion of the *BAR1* gene causes **a**‐cells to become hypersensitive towards the α‐factor pheromone [35, 36]. The protein Far1p (“factor arrest”) plays a crucial role in the cell cycle arrest of haploid yeast cells. It acts as inhibitor of the cyclin‐dependent kinase Cdc28p, which forms a complex with the cyclins Cln1/2p in order to regulate the transition from G1 to S phase [37, 38]. Far1p is further involved in the polarized growth of the yeast cell during mating [39]. For our studies, we worked with an *S. cerevisiae* MAT**a** strain possessing a deletion of its *BAR1* and *FAR1* genes (Δ*bar1*Δ*far1*) in order to gain high sensibility to α‐factor and avoid growth arrest upon α‐factor presence.

Met15p (synonymous with Met17p, Met25p [40, 41, 42]) is an O‐acetylhomoserine sulfhydrolase catalyzing the terminal biosynthetic step of the yeast sulfate assimilation pathway (SAP) [43] by generating homocysteine from O‐acetyl‐L‐homoserine and hydrogen sulfide [42, 44, 45]. Homocysteine is further converted either directly to methionine or indirectly to cysteine. Both sulfur amino acids are required for yeast growth [43]. Deletion of *MET15* in *S. cerevisiae* (*met15*Δ*0*) is known as a classic auxotrophic selection marker, causing cells to suffer from a lack of organosulfur compounds like methionine, cysteine or homocysteine [46, 47]. Recent studies explained the growth failure of *met15*Δ*0* mutants by the accumulation of toxic levels of hydrogen sulfide due to a metabolic bottleneck [48]. For simplicity, we will use the classic term “auxotrophic selection marker” in this article when referring to the *met15*Δ*0* mutant.

In this work, we aimed on generating genetic modules for a controllable, α‐factor‐dependent growth behavior of yeast cells. Different expression vectors carrying genetic modules consisting of either *MET15* or *FAR1* under control of the *FIG1* promoter were created. Growth restoration in dependence of increasing α‐factor concentrations should be achieved by *MET15* expression and the tiered complementation of the methionine auxotrophy of the *S. cerevisiae* MAT**a** Δ*bar1*Δ*far1* strain (*met15*Δ*0*) (Figure 1(A)). In contrast, the inhibitory effect of Far1p on the cell cycle should result in a gradual reduction of yeast growth with increasing α‐factor‐concentration although the strain possesses a deletion of its *FAR1* gene (Figure 1(B)). Fine‐tuning of the growth should be possible by using expression plasmids with different copy numbers, as well as addition of different concentrations of α‐factor resulting in graduated expression of the target genes and hence controlled growth rates.

## 2 MATERIALS AND METHODS

### 2.1 Strains and media

*Saccharomyces cerevisiae* wild‐type strain BY4742 (Acc. no. Y10000) and deletion strain BY4741 Δ*bar1* (Acc. no. Y01408) were ordered from Euroscarf. *S. cerevisiae* BY4741 Δ*bar1*Δ*far1* (MAT**a** *his3*Δ*1 leu2*Δ*0 met15*Δ*0 ura3*Δ*0* YIL015w::kanMX4 YJL157c::natMX6) was kindly provided by Dr. Stefan Hennig (Faculty Biology, TU Dresden). *S. cerevisiae* wild‐type strain as well as BY4741 Δ*bar1*Δ*far1* were cultured in full medium (10 g/L yeast extract, 20 g/L peptone plus 20 g/L glucose (YPD) or 30 g/L glycerol (YPG)) at 30°C. Recombinant strains were grown in minimal medium (1.7 g/L yeast nitrogen base, 5.0 g/L ammonium sulfate, 20 g/L glucose) supplemented with l‐histidine (60 mg/L) and l‐leucine (80 mg/L) for the cultivation of strains carrying *MET15* plasmid based genetic modules, and additionally l‐methionine (20 mg/L) for *FAR1* expression strains. *Escherichia coli* TOP10 cells (Thermo Fisher Scientific) were cultivated in LB medium (10 g/L peptone, 5 g/L yeast extract, 5 g/L sodium chloride) at 37°C. If required, ampicillin (100 mg/L) was added to the medium.

### 2.2 General DNA methods

Genomic DNA from *S. cerevisiae* was extracted with the Yeastar Genomic DNA Kit (Zymo Research). Extraction of DNA fragments from agarose gels was done using the Zymoclean Gel DNA Recovery Kit (Zymo Research). For restriction digestions, the CutSmart Buffer system and respective endonucleases (New England Biolabs) were used. Ligation of DNA was achieved using a T4 DNA ligase (Thermo Fisher Scientific). Plasmid propagation was performed in *E. coli* TOP10 using standard protocols. Plasmid DNA was isolated from *E. coli* with the ZR Plasmid MiniprepTM‐Classic Kit (Zymo Research).

### 2.3 Construction of *MET15* and *FAR1* expression plasmids

For the generation of *MET15* and *FAR1* expression plasmids, the *S. cerevisiae* vectors p416 (low copy number, *CEN/ARS*) or p426 (high copy number, 2*μ*) have been used as backbone [49]. Promoter *FIG1* was cloned into plasmids p416 or p426, giving rise to plasmids p416FIG1 [kindly provided by Dr. Stefan Hennig] and p426FIG1 [32], respectively. The target genes *MET15* (YLR303W; 1335 bp) and *FAR1* (YJL157C; 2493 bp) were PCR‐amplified from genomic DNA of *S. cerevisiae* BY4742 using a Phusion High‐Fidelity DNA Polymerase (Thermo Fisher Scientific) and following primers: MET15_NheI_for and MET15_XhoI_rev for *MET15*, and primers FAR1_AvrII_for and FAR1_XhoI_rev for *FAR1* (Table S1). Amplicons were ligated into the corresponding restriction sites of plasmids p416FIG1 and p426FIG1, leading to the expression vectors p416FIG1‐MET15, p426FIG1‐MET15, p416FIG1‐FAR1 and p426FIG1‐FAR1, respectively. Further, the nucleotide sequence coding for the glycine serine (Gly_4_Ser)_3_ linker and three copies of the hemagglutinin tag (HA_3_‐tag) was ligated downstream of *MET15* or *FAR1*, respectively, giving rise to following expression vectors designed for the production of HA_3_‐tagged proteins: p416FIG1‐MET15‐HA_3_, p426FIG1‐MET15‐HA_3_, p416FIG1‐FAR1‐HA_3_ and p426FIG1‐FAR1‐HA_3_. All plasmids used in this study are summarized in Table S2. Expression plasmids were transformed into *S. cerevisiae* BY4741 Δ*bar1*Δ*far1* using the lithium acetate/single‐stranded carrier DNA/polyethylene glycol method [50].

### 2.4 Expression analysis of recombinant proteins

For the production of Met15p‐HA_3_ and Far1p‐HA_3_, shaken flask cultures were inoculated with overnight cultures of the expression strains to an OD_600_ of 1.0. Immediately, expression was initiated by the addition of synthetic α‐factor (Molsurf) to a final concentration of 0.25 µM. Previous experiments with increasing concentrations of α‐factor for induction found that 0.25 µM is the optimal concentration for the best detectable protein bands (data not shown). An uninduced culture was run in parallel. Expression analysis was conducted at 30°C under constant agitation at 180 rpm. Samples were taken 0, 2, 4, 6 and 24 h after induction and cells were harvested by centrifugation at 3500 × *g* for 5 min. Subsequently, cells were washed with distilled sterile water, suspended in phosphate‐buffered saline containing protease inhibitor cocktail (Roche) and disrupted mechanically by the addition of glass beads and shaking in a mixer mill (Retsch) for 5 min at 30 Hz. Protein concentration of the supernatant was determined using the DC Protein‐Assay (Bio‐Rad).

### 2.5 SDS‐PAGE, Coomassie staining and Western blot analysis

Proteins (20 µg per lane) were separated by SDS‐PAGE according to Laemmli [51] using 5% and 10% acrylamide concentration for stacking and running gel, respectively. Proteins were transferred from the polyacrylamide gel to an Immobilon‐P polyvinylidene fluoride membrane (0.45 µm; Merck Millipore) using Western blot, probed with primary antibodies directed against the HA_3_‐tag (Roche) and detected with horseradish peroxidase‐marked secondary antibodies (GE Healthcare) and the WesternBright Kit (Biozym Scientific). Coomassie staining of the proteins in the polyacrylamide gel was performed according to Neuhoff et al. [52].

### 2.6 Halo assay

To visualize the positive or negative effect of the expression of *MET15* or *FAR1* on the growth of *S. cerevisiae*, a modified halo assay was performed [53]. Shaken flask cultures of the *S. cerevisiae* BY4741 Δ*bar1*Δ*far1 MET15* or *FAR1* expression strains were inoculated in minimal medium. After a cultivation time of 4 h, cells were harvested (3500 × *g*, 5 min), washed and suspended in distilled water. Cell suspensions were adjusted to an OD_600_ of 0.001 and 100 µL plated on minimal medium agar plates. Subsequently, sterile filter platelets were placed centered on the plates and soaked with 5 µL synthetic α‐factor (1.0 µg/µL) or double‐distilled sterile water (negative control), respectively. After 96 h incubation, growth of *S. cerevisiae* on the agar plates was evaluated.

### 2.7 Growth analysis by nephelometry

Precultures of *S. cerevisiae* BY4741 Δ*bar1*Δ*far1 MET15* and *FAR1* expression strains were grown in minimal medium (supplemented with required supplements). Cells were harvested, washed with distilled water and suspended to a concentration of 1.25 × 10^6^ cells per mL. 200 µL of the respective minimal medium was inoculated with 10^4^ cells per well in a 96‐well plate. Growth of the yeast cells was monitored using the NepheloStar (BMG LABTECH) under following conditions: temperature: 30°C, shaking: 170 rpm, width 3 mm, orbital; cycles: 200, 30 min each. Each strain was monitored in two independent experiments three times with the NepheloStar, resulting in each curve derived from six data sets (*n* = 6). The growth rate in the exponential phase of the control (0 µM α‐factor) was calculated and compared with the values of the other α‐concentrations by unpaired *t*‐test. Differences were considered as significant with a *p*‐value ≤ 0.05 (*p ≤ 0.05, **p ≤ 0.01, ***p ≤ 0.005, ****p ≤ 0.001).

## 3 RESULTS

### 3.1 α‐Factor pheromone‐dependent expression of *MET15* and *FAR1* in recombinant *S. cerevisiae* strains

Aiming on an α‐factor‐dependent expression of *MET15* and *FAR1*, the genes were set under control of the α‐factor sensitive *FIG1* promoter and cloned in either the low copy number p416 or the high copy number p426 vector (Table S2). Additionally, for both target proteins also versions carrying an HA_3_ epitope tag were generated to facilitate subsequent detection of the proteins using HA‐specific antibodies. All expression vectors were transformed into *S. cerevisiae* BY4741 Δ*bar1*Δ*far1* strain.

**FIGURE 2:**
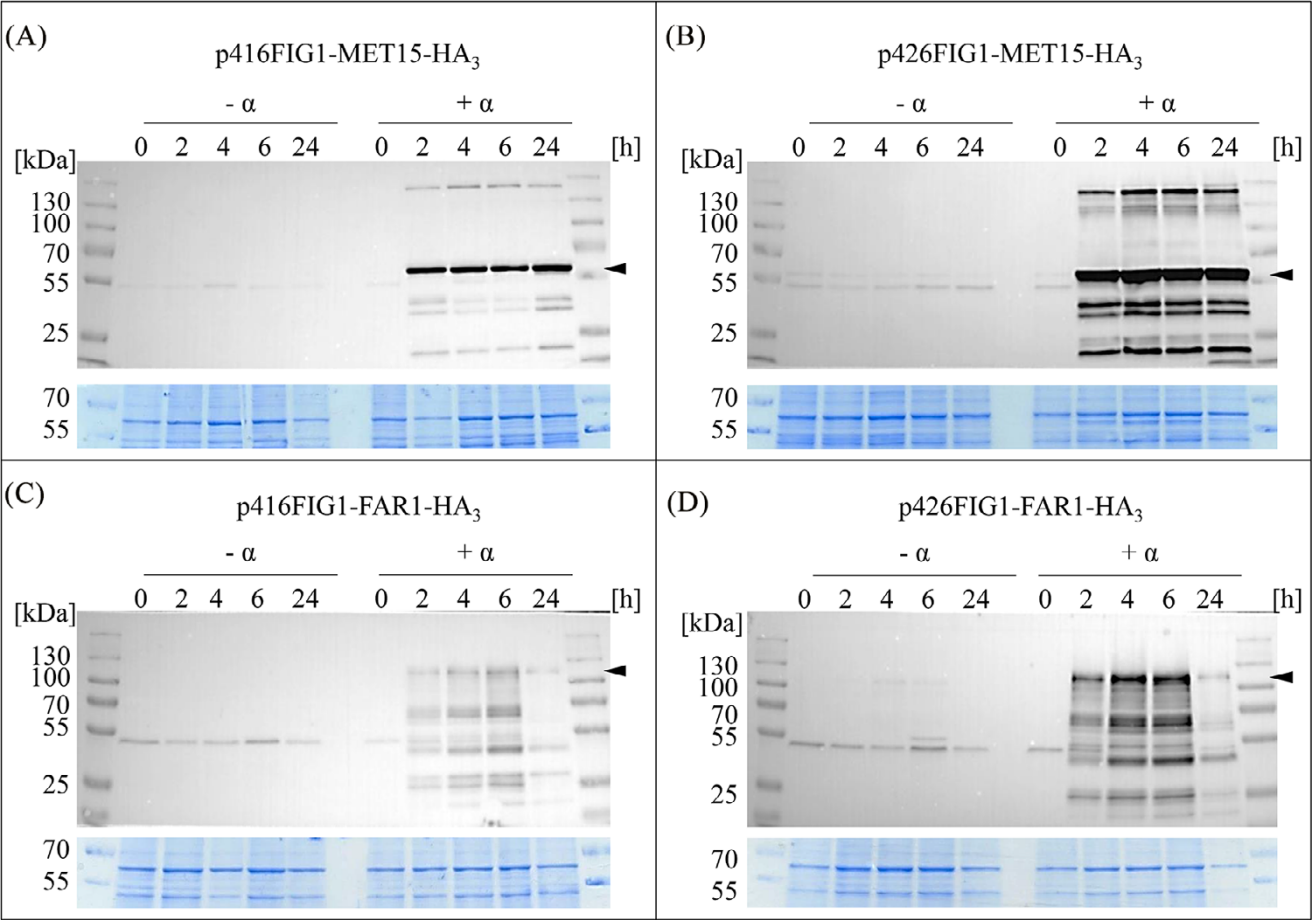
Expression analysis of *S. cerevisiae* BY4741 Δ*bar1*Δ*far1* carrying plasmid p416FIG1‐MET15‐HA_3_ (A), p426FIG1‐MET15‐HA_3_ (B), p416FIG1‐FAR1‐HA_3_ (C) or p426FIG1‐FAR1‐HA_3_ (D). Strains were cultivated for 24 h without (‐ α) or with 0.25 µM synthetic α‐factor (+ α). Soluble protein fractions were separated in a 10% SDS‐PAGE and Western blot analysis was performed using HA_3_‐specific antibodies. The arrow indicates the recombinant target proteins Met15p‐HA_3_ (Mw calc. ∼ 53 kDa) and Far1p‐HA_3_ (Mw calc. ∼ 99 kDa), respectively. Total protein amount was visualized in the protein gels by colloidal Coomassie staining (lower panels).

As a prerequisite for the functionalization of the constructed *MET15* and *FAR1* plasmids as growth control modules, the inducible expression of the respective recombinant proteins was evaluated. For this purpose, strains expressing the HA_3_‐tagged versions of *MET15* or *FAR1* were cultivated with and without the addition of 0.25 µM synthetic α‐factor pheromone. Samples of the yeast cultures were taken after 0, 2, 4, 6, and 24 h and the soluble protein fraction of each sample was analyzed by SDS‐PAGE and Western blot (Figure 2).

In the soluble fraction of *MET15‐HA_3_* expressing *S. cerevisiae* cells, already 2 h after induction a prominent protein band sizing ∼55 kDa occurred, consistent with the calculated molecular weight (Mw calc.) of recombinant Met15p‐HA_3_ of approximately 53 kDa (Figure 2A,B, arrow). The intensity of the protein signal remained constant for at least 24 h. The protein band appeared stronger when expression was performed with the high copy number plasmid p426, presumably due to higher expression levels (Figure 2B). In the induced fractions, further protein bands sensitive towards the HA‐specific antibody have been observed, which most likely derived from multimeric protein variants in the case of bands at higher molecular weight or proteolytic degradation products exhibiting a lower molecular weight, respectively.

Soluble fractions of *S. cerevisiae FAR1‐HA_3_* expression strains contained a characteristic protein band in the range of 100 to 130 kDa, which also occurred 2 h after induction with α‐factor (Figure 2C,D, arrow). The Mw calc. of recombinant Far1p‐HA_3_ is about 99 kDa. The difference between the calculated and the apparent molecular weight of Far1p‐HA_3_ might be due to posttranslational modifications of the protein. In vivo, Far1p is activated by phosphorylation in response to the α‐factor pheromone, causing a shift in its mobility as reported previously [54]. Additionally, high amount of acidic amino acid residues can influence the migration of a protein on SDS‐PAGE [55], possibly resulting in an increased apparent molecular weight of Far1p‐HA_3_. Detectable protein bands with lower molecular weight most likely represent degradation products of the target protein. The signal intensity of all protein bands decreased in the induced fractions after 6 h of cultivation. As already observed for *MET15*, the usage of the high copy number p426 plasmid resulted in enhanced signal strength compared to the low copy number plasmid.

All samples in all expression strains contained a protein signal of ∼50 kDa in the Western blot analysis, regardless of whether the expression was α‐factor induced or not, indicating that these are not the target proteins Met15p‐ HA_3_ or Far1p‐HA_3_ (Figure 2). Apparently, this is a nonspecific cross‐reaction of the HA‐antibody with another soluble protein of *S. cerevisiae* under the used culture conditions, as it was also visible in wild‐type samples as well as the host strain Δ*bar1*Δ*far1* when cultured in minimal medium (Figure S1).

### 3.2 α‐Factor‐dependent expression of *MET15* allows fine tuning growth rates in *MET15*‐expression strains

**FIGURE 3:**
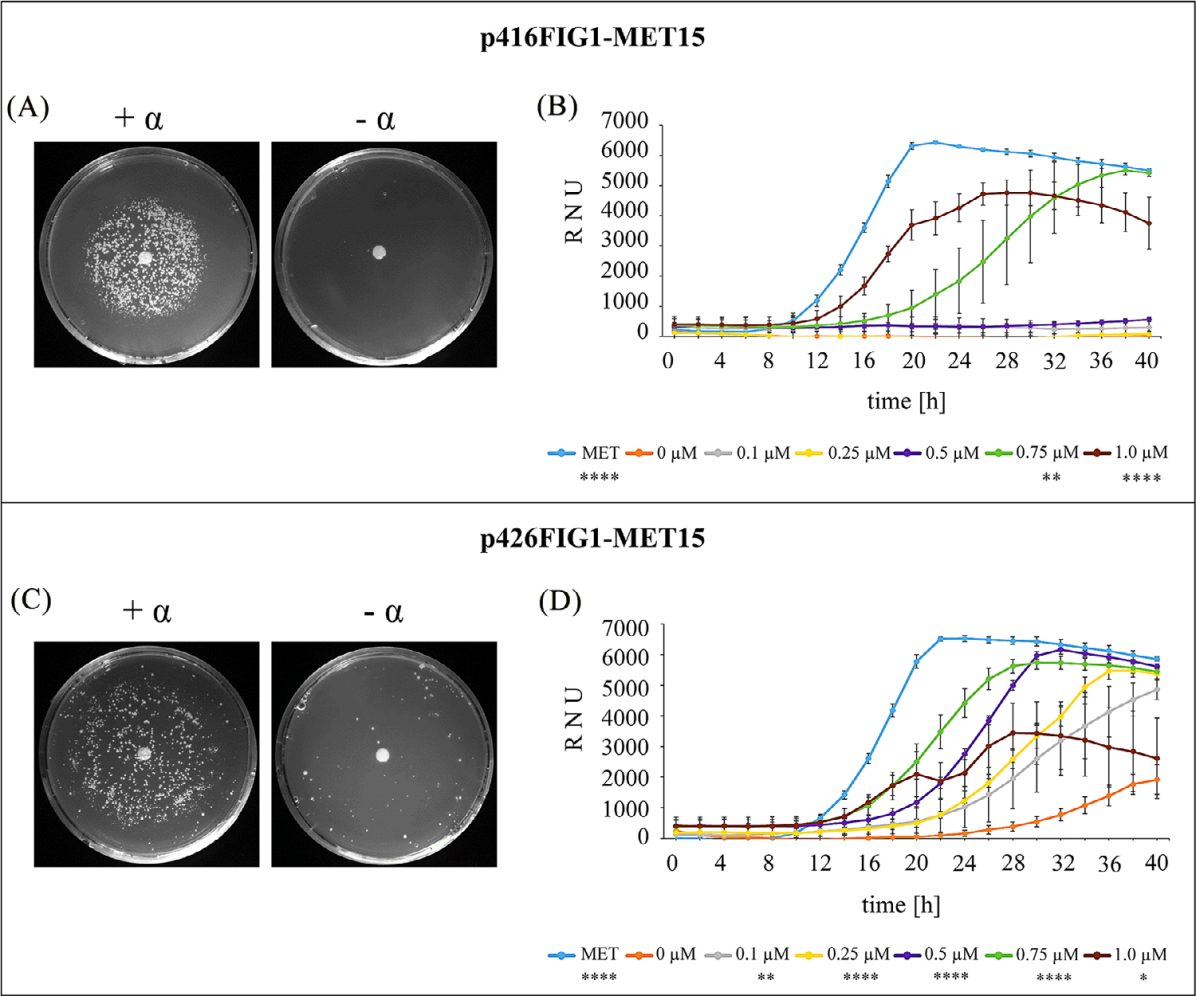
α‐Factor dependent growth rescue of methionine auxotroph *S. cerevisiae FIG1‐MET15* expression strains. Yeasts carrying p416 low copy number plasmid (A, B) as well as the p426 high copy number plasmid (C, D) were analyzed. For the halo assay (A, C), the filter paper was either soaked with 5 µg of synthetic α‐factor (+ α) or with water (‐ α). Plates were incubated for 96 h before evaluation. For the nephelometric measurements (B, D), growth of the strains was monitored for 40 h after the addition of different α‐factor concentrations (0 µM to 1.0 µM). As positive control, methionine was added directly to the medium (MET). Curves represent mean values (*n* = 6) and error bars indicate standard deviations (± SD). Growth rates were compared with the values of the control (0 µM α‐factor) by unpaired *t*‐test and are marked if significant (*p ≤ 0.05, **p ≤ 0.01, ***p ≤ 0.005, ****p ≤ 0.001).

The methionine‐dependent *S. cerevisiae* BY4741 Δ*bar1*Δ*far1* (*met15*Δ*0*) strain was transformed with *MET15* expression plasmid p416FIG1‐MET15 or p426FIG1‐MET15, respectively. To demonstrate that the recombinant Met15p protein is biologically active and restores growth of the methionine auxotrophic *S. cerevisiae* Δ*bar1*Δ*far1* (*met15*Δ*0*) strain, a halo assay was performed (Figure 3A,C). *MET15* expression strains were cultured on agar plates without methionine and equipped with a filter paper soaked with synthetic α‐factor pheromone (+ α) or, as negative control, with distilled sterile water (‐ α). After 96 h of incubation, a lawn of colonies was growing on the α‐factor positive plates (+ α), spreading in a circle around the centered filter. Higher expression of MET15p in p426‐MET15 strain enabled growth of colonies with a further distance to the center than observed for colonies carrying the low copy number p416FIG1‐MET15 plasmid. Interestingly, using p426FIG1‐MET15 resulted in the growth of some colonies on agar plates lacking α‐factor (Figure 3C, ‐ α). A reason for the growth of some colonies in the negative control could be due to a slight background expression of *MET15* under high copy number conditions.

Subsequently, growth of the *MET15* expression strains after induction with different α‐factor concentrations was monitored by nephelometric measurements (Figure 3B,D). As negative control for the nephelometer analysis, growth of strains carrying the empty vectors (p416FIG1 or p426FIG1) was measured showing that the addition of the used α‐factor concentrations did not have any remarkable influence on growth (Figure S2). As positive control, l‐methionine was added directly to the medium, providing the possibility to measure growth of the yeast under nonauxotrophic nutritional conditions. Optimal growth entered exponential phase at around 12 h cultivation and reached a maximum after 20 to 22 h with approx. 6500 relative nephelometric units (RNU). When cultivation was conducted without methionine and α‐factor (negative control, 0 µM), no growth of *S. cerevisiae* was observable due to the methionine auxotrophy. When using the low copy number plasmid p416FIG1‐MET15 for expression (Figure 3B), a concentration of 0.75 µM α‐factor was required to create a measurable effect of Met15p on the growth of *S. cerevisiae* and beginning of the exponential phase was delayed with a reduced maximum RNU compared to the positive control. Induction with 1.0 µM α‐factor in p416‐MET15 resulted in a slightly delayed entering to the stationary phase compared to the positive control, with a reduced overall growth and a maximum of about 5000 RNU after 24 h. Growth analysis of the strain carrying high copy number plasmid p426FIG1‐MET15 (Figure 3D) revealed a slight growth of the negative control after 22 h of cultivation. Consistent with the observation in the halo assay, some cells of the *MET15* expression strain also grow without α‐factor induction, but only when expression vector p426 was used and cultivation exceeded 22 h. A concentration of 0.25 µM α‐factor restored the growth, with delayed exponential phase and overall growth reduction, as observed for p416‐based expression in the presence of 0.75 µM α‐factor. The results clearly show a correlation between the added α‐factor concentration and growth, whereby lower α‐factor concentrations are sufficient to exceed the threshold of methionine synthesis in the high copy number version.

### 3.3 Far1p initiated growth stop of *S. cerevisiae* induced by α‐factor pheromone

**FIGURE 4:**
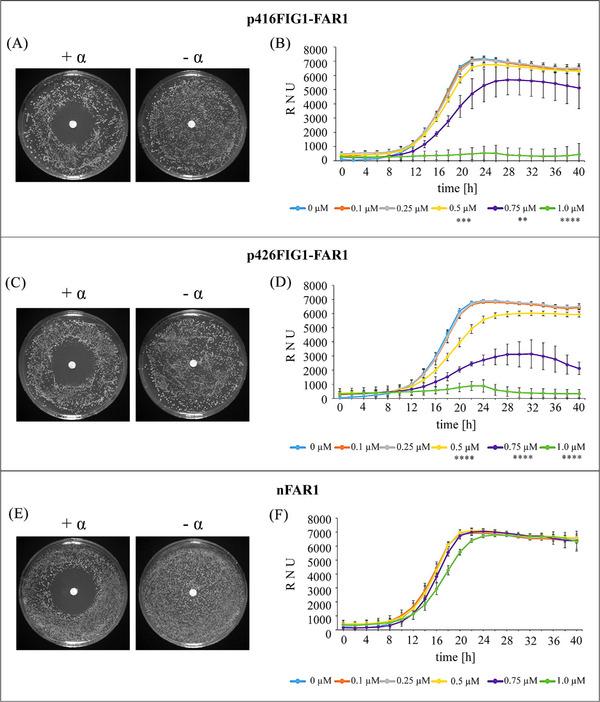
α‐Factor‐dependent *FAR1* induced growth inhibition of *S. cerevisiae*. Growth of the *FIG1‐FAR1* expression strains carrying the p416 low copy number plasmid (A, B), or the p426 high copy number plasmid (C, D), as well as a control strain possessing a functional, natural *FAR1* gene (E, F) was analyzed. Halo assays (A, C, E) were performed using either 5 µg of synthetic α‐factor (+ α) or water (‐ α). Plates were incubated for 96 h before evaluation. For the nephelometric measurements (B, D, F), growth of the strains was monitored for 40 h after the addition of different α‐factor concentrations (0 µM to 1.0 µM). Curves represent mean values (*n* = 6) and error bars indicate standard deviations (± SD). Growth rates were compared with the values of the control (0 µM α‐factor) by unpaired *t*‐test and are marked if significant (*p ≤ 0.05, **p ≤ 0.01, ***p ≤ 0.005, ****p ≤ 0.001).

The functionality and effect of recombinant Far1p on the growth of *S. cerevisiae* p416FIG1‐FAR1 or p426FIG1‐FAR1 expression strains was as well evaluated using the halo assay (Figure 4A,C). As control, growth of *S. cerevisiae* BY4741 Δ*bar1* possessing the natural *FAR1* (“*nFAR1*”) gene but lacking any expression plasmid, was tested (Figure 4E). After 96 h incubation, a distinctive growth inhibition area was visible on the agar plates carrying a centered filter paper containing α‐factor (+ α), whereby growth was not affected in the negative control (‐ α). The α‐factor concentration required for sufficient expression of *FAR1* was exceeded, regardless of whether it was plasmid‐based *FIG1*‐controlled or native expression.

Further, growth of the *FIG1‐FAR1* expression strains induced with increasing α‐factor concentrations was monitored by nephelometric measurements (Figure 4B,D). The blue line represents growth in the absence of α‐factor (0 µM), serving as positive control. Already the addition of 0.5 µM α‐factor led to a reduction of the yeast growth, whereby the effect was more distinct in the strains carrying the high copy number plasmid p426. A concentration of 0.75 µM α‐factor delayed and minimized the logarithmic phase of the yeast growth and 1.0 µM α‐factor even hampered the growth completely independent of the plasmid copy number. Growth of the *S. cerevisiae* BY4741 Δ*bar1* strain with natural *FAR1* (“*nFAR1*”) expression was not affected up to 0.75 µM α‐factor (Figure 4F). Addition of 1.0 µM α‐factor slightly influenced growth, indicated by a delayed logarithmic phase, but not led to a growth arrest, as it is the case for *S. cerevisiae* Δ*bar1*Δ*far1 FAR1* expression strains. The artificial control of *FAR1* expression by the *FIG1* promoter in the *S. cerevisiae* Δ*bar1*Δ*far1* strains carrying p416FIG1‐FAR1 or p426FIG1‐FAR1, respectively, leads to a much higher sensitivity towards the α‐factor pheromone compared to the expression of the natural *nFAR1* regulated by its native promoter.

## 4 DISCUSSION

The aim of our study was to generate genetic modules that allow precise and targeted α‐factor pheromone‐dependent growth control of *S. cerevisiae*. To this end, a set of different plasmid‐based modules have been constructed, carrying the target genes *MET15* or *FAR1* under control of the α‐factor sensitive *FIG1* promoter either in the p416 low copy number or the p426 high copy number plasmid backbone.

We observed a direct influence of the α‐factor pheromone concentration on the *MET15* and *FAR1* target gene expression. The suitability of the *FIG1* promoter to control gene expression by the addition of α‐factor pheromone is already known [31, 32]. However, we also modulated the expression levels of *MET15* and *FAR1* by using two different vectors (p416, p426 [49]) with low and high copy numbers, respectively, and increasing concentrations of α‐factor pheromone for induction, which in turn allows a precise fine‐tuning of growth behavior.

Auxotrophic *S. cerevisiae met15*Δ*0* strain harboring the *FIG1‐MET15* genetic module recovered growth in the presence of α‐factor (Figure 3). Vice versa, the genomic *far1* deletion (Δ*far1*), which leads to abolition of the yeast cell cycle arrest, was restored by the plasmid‐based *FIG1* controlled expression of *FAR1* and caused growth decrease with increasing α‐factor concentrations added (Figure 4). It is conceivable that other genes could be added to our genetic modules to modulate growth behavior, for example, by the controlled expression of lethal restriction enzymes.

The natural *S. cerevisiae* pheromone response system provides an excellent tool for manipulating and targeting yeast communication. Instead of the synthetic α‐factor used in our study, cellular produced pheromones in a co‐culture could be used as inducers of the *FIG1* promoter. For example, an artificial, blue light‐dependent and α‐factor pheromone mediated communication between two *S. cerevisiae* strains was reported recently [56]. Thereby, activation of the sender cell under blue light conditions led to the secretion of α‐factor, which induced the mating pathway in the receiver cell through binding at the Ste2p GPCR. Consequently, the transcriptional luciferase reporter gene in the receiver cell, placed under control of an α‐factor inducible promoter, was expressed.

However, it would also be possible to use the generated yeast strains for a co‐cultivation with another microorganism capable of heterologous production of recombinant α‐factor. This would allow artificial communication between yeast and another organism coupled to internal yeast growth control with high specificity to *S. cerevisiae* **a‐**cells. A targeted, pheromone‐based interspecies communication between *S. cerevisiae* and the yeast *Schizosaccharomyces pombe* has already been established [31]. A programmed cross‐kingdom communication between *E. coli* and *S. cerevisiae* using a nanoparticle as translator between both microorganisms to decode the otherwise incomprehensible chemical messages was also reported [57]. Direct communication between *S. cerevisiae* and another prokaryote, controlled by the heterologous production of the corresponding pheromones, would also be conceivable and is part of our ongoing projects. The use of yeast with controllable growth behavior would be a decisive advantage in such co‐cultures. Using α‐factor is highly specific for *S. cerevisiae* **a‐**cells, as only these possess the Ste2p GPCRs on their cell surface [10], so that the risk of secondary reaction of the other organism can be minimized with this approach.

In addition to the aspect of artificial communication, a controllable co‐cultivation of *S. cerevisiae* with another biotechnologically relevant microorganism offers many benefits in terms of production efficiency [58]. Sharing the biosynthetic performance between two host organisms reduces the metabolic burden on the individual organism and leads to a higher metabolic efficiency compared to monocultures. In addition, mixed populations can benefit from the different nutritional requirements, for example, allowing unwanted by‐products to be reused by another strain rather than accumulating [59, 60]. A balanced growth of both microorganism populations in co‐cultures represents a substantial challenge. Therefore, the yeast strains generated in this work represent ideal partners for co‐cultivation due to their adjustable growth rate.

## CONFLICT OF INTEREST STATEMENT

The authors have declared no conflicts of interest. The manuscript does not include animal experiments or human studies.

## Supporting information



Supporting Information

## Data Availability

The data that support the findings of this study are available from the corresponding author upon reasonable request.
