# Supplementary material for: Genetic modules for α‐factor pheromone controlled growth regulation of Saccharomyces cerevisiae
Source: Eng Life Sci. 2024 May 22;24(8):e2300235. doi: 10.1002/elsc.202300235 (PMC11300815; doi:10.1002/elsc.202300235)
Supplement: Supplementary file 1 — Supporting Information [file ELSC-24-e2300235-s001.docx]

**Supporting information**

Table S1. Primers and nucleotide sequences used in this study

| **Primer** | **Sequence (5’ – 3’)** | **Description** |
| --- | --- | --- |
| FAR1_AvrII_for | TCGTCTCCTAGGATGAAGACACCAACAAGAGTTTCGTTTG | *FAR1* forward primer carrying *Avr*II restriction site |
| FAR1_XhoI_rev | TGCACTCTCGAGCTAGAGGTTGGGAACTTCCAGGGTC | *FAR1*  reverse primer carrying *Xho*I restriction site |
| MET15_NheI_for | AGCTGAGCTAGCATGCCATCTCATTTCGATACTGTTCAAC | *MET15* forward primer carrying *Nhe*I restriction site |
| MET15_XhoI_rev | TAGTCTCTCGAGTCATGGTTTTTGGCCAGCGAAAAC | *MET15* reverse primer carrying *Xho*I restriction site |

Table S2. Plasmids used in this study

| **Plasmid** | **Relevant properties** | **Source or reference** |
| --- | --- | --- |
| p416FIG1 | *S. cerevisiae* low copy number plasmid (*CEN/ARS*)  *URA3* selection marker  α-factor inducible promoter P*_FIG1_*  CYC1 terminator | [32, 49] |
| p416FIG1-FAR1 | p416-based FAR1 expression plasmid | This study |
| p416FIG1-FAR1-HA_3_ | p416-based FAR1-HA_3_ expression plasmid | This study |
| p416FIG1-MET15 | p416-based MET15 expression plasmid | This study |
| p416FIG1-MET15-HA_3_ | p416-based MET15-HA_3_ expression plasmid | This study |
| p426FIG1 | *S. cerevisiae* high copy number plasmid (2*µ*)  *URA3* selection marker  α-factor inducible promoter P*_FIG1_*  CYC1 terminator | [32, 49] |
| p426FIG1-FAR1 | p426-based FAR1 expression plasmid | This study |
| p426FIG1-FAR1-HA_3_ | p426-based FAR1-HA_3_ expression plasmid | This study |
| p426FIG1-MET15 | p426-based MET15 expression plasmid | This study |
| p426FIG1-MET15-HA_3_ | p426-based MET15-HA_3_ expression plasmid | This study |

**
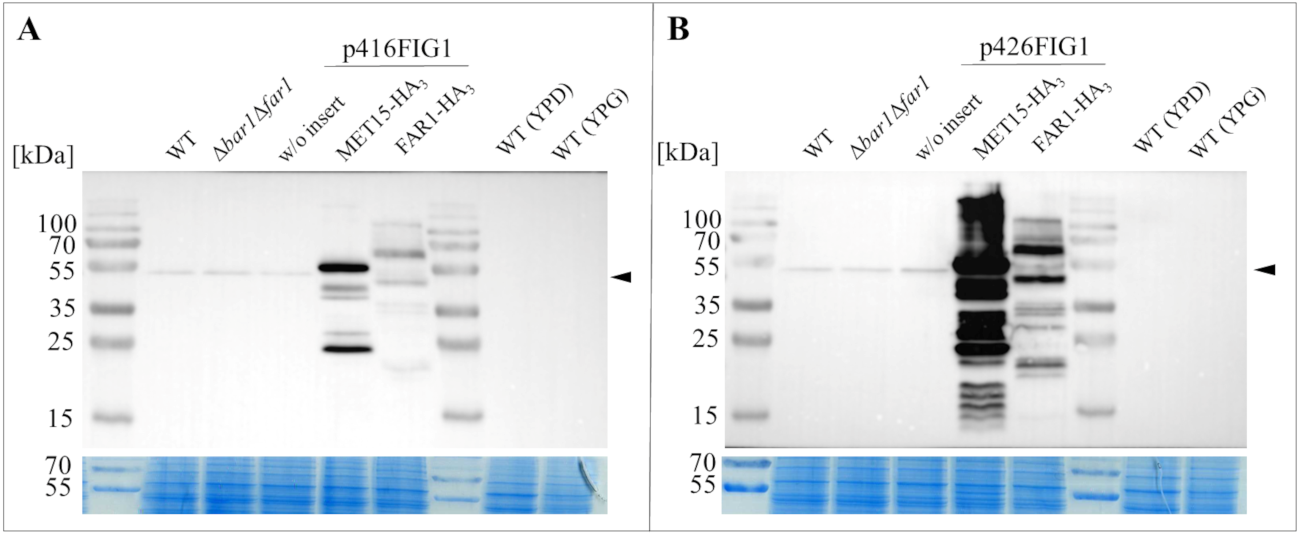
**

**Figure S1:** Expression analysis of *S. cerevisiae* BY4741 (WT), Δ*bar1*Δ*far1*, and Δ*bar1*Δ*far1* carrying the respective low copy (p416) or high copy (p426) plasmids. Strains were cultivated in minimal medium for 4 h with 0.25 µM synthetic α-factor or full medium (YPD or YPG). Cells were disrupted, soluble protein fractions (20 µg per lane) were separated in a 10 % SDS-PAGE and Western blot analysis was performed using HA_3_-specific antibodies. The arrow indicates the position of the unspecific protein band of about ~ 50 kDa visible in all samples cultured on minimal medium, but not in full medium. Total protein amount in the protein gels was visualized by colloidal Coomassie staining (lower panels).


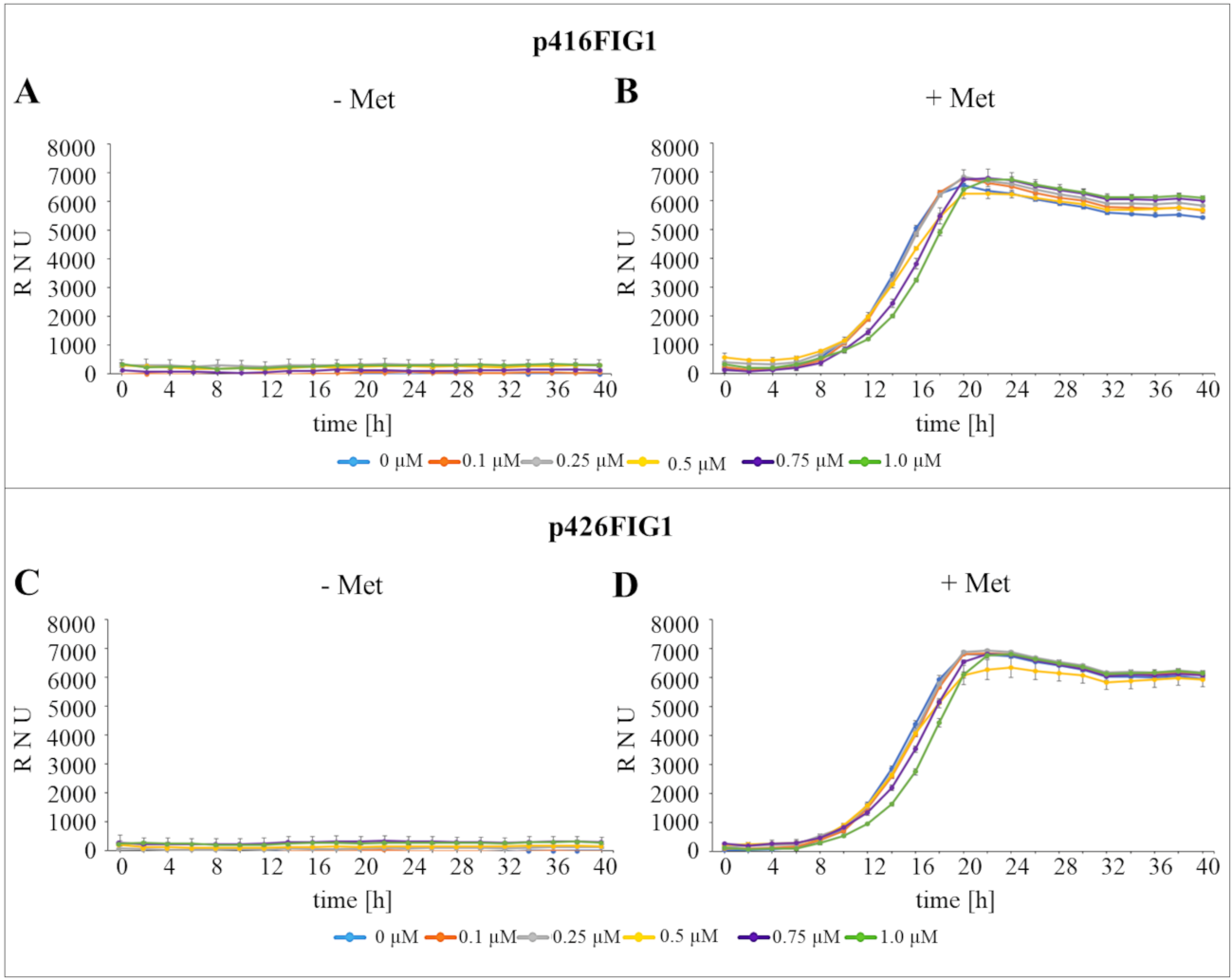


**Figure S2: N**ephelometric measurements of *S. cerevisiae* strains carrying the p416FIG1 low copy number plasmid (A, B) or the p426FIG1 high copy number plasmid (C, D) containing only the promotor *FIG1* without any insert. Growth of the strains was monitored for 40 h after the addition of different α‑factor concentrations (0 µM to 1.0 µM) in minimal medium with and without the supplementation of methionine. Curves represent mean values from a single experiment measured in triplicates (+/‑ SD). Growth rates were compared with the values of the control (0 µM α-factor) by unpaired t-test (no significant changes).
